# Prognostic impact of alkaline phosphatase measured at time of presentation in patients undergoing primary percutaneous coronary intervention for ST-segment elevation myocardial infarction

**DOI:** 10.1371/journal.pone.0171914

**Published:** 2017-02-09

**Authors:** Pyung Chun Oh, Kyounghoon Lee, Tae-Hoon Kim, Jeonggeun Moon, Hyun Woo Park, Ho-Jun Jang, Sang-Don Park, Sung Woo Kwon, Jon Suh, Woong Chol Kang

**Affiliations:** 1 Cardiology, Gachon University Gil Medical Center, Incheon, Republic of Korea; 2 Cardiology, Sejong General Hospital, Bucheon, Republic of Korea; 3 Cardiology, Soon Chun Hyang University Bucheon Hospital, Bucheon, Republic of Korea; 4 Cardiology, Inha University Hospital, Incheon, Republic of Korea; Azienda Ospedaliero Universitaria Careggi, ITALY

## Abstract

**Background:**

Serum alkaline phosphatase (ALP) has been shown to be a prognostic factor in several subgroups of patients due to its promotion of vascular calcification. However, the prognostic impact of serum ALP level in ST-segment elevation myocardial infarction (STEMI) patients with a relatively low calcification burden has not been determined. We aimed to investigate the association of ALP level measured at time of presentation on clinical outcomes in patients with STEMI requiring primary percutaneous coronary intervention (PCI).

**Methods:**

A total of 1178 patients with STEMI undergoing primary PCI between 2007 and 2014 were retrospectively enrolled from the INTERSTELLAR registry and classified into tertiles by ALP level (<64, 65–82, or >83 IU/L). The primary study outcome was a major adverse cardiac or cerebrovascular event (MACCE), defined as the composite of all-cause death, non-fatal myocardial infarction, non-fatal stroke, and ischemia-driven revascularization.

**Results:**

Median follow-up duration was 25 months (interquartile range, 10–39 months). The incidence of MACCE significantly increased as ALP level increased, that is, for the <64, 65–82, and >83 IU/L tertiles incidences were 8.7%, 11.7%, and 15.7%, respectively; p for trend = 0.003). After adjustment for potential confounders, the adjusted hazard ratios for MACCE in the middle and highest tertiles were 1.69 (95% CI 1.01–2.81) and 2.46 (95% CI 1.48–4.09), respectively, as compared with the lowest ALP tertile.

**Conclusions:**

Elevated ALP level at presentation, but within the higher limit of normal, was found to be independently associated with higher risk of MACCE after primary PCI in patients with STEMI.

## Introduction

Calcific vasculopathy is one of the major contributors to atherosclerosis, and causes vascular hardening, aging, and finally, adverse cardiovascular events [1–3]. The development of calcification in atherosclerotic lesions is the result of osteogenic differentiation in subpopulations of vascular cells exposed to various stimuli [4]. Alkaline phosphatase (ALP) catalyzes the hydrolysis of organic pyrophosphate, a potent inhibitor of vascular calcification, and thus, promotes calcification [5]. Serum ALP level has been shown to be a prognostic indicator of future cardiovascular events and mortality in patients with chronic kidney disease [6, 7], and an elevated ALP level, perhaps due to its promotion of vascular calcification, has been shown to be associated with increased risk of cardiovascular events in the general population with normal kidney function and in survivors of stroke or myocardial infarction (MI) [8–10]. However, the prognostic impact of serum ALP level in patients with acute MI requiring primary percutaneous coronary intervention (PCI) has not been determined. Thus, the purpose of this study was to evaluate the hypothesis that higher levels of ALP at time of presentation are associated with an increased risk of adverse cardiovascular events in patients with ST-segment elevation MI (STEMI) requiring primary PCI.

## Methods

### Study population

The analysis was conducted using data obtained from the “INcheon-Bucheon cohorT of patients undERgoing primary PCI for acute ST-ELevation myocardiaL infARction (INTERSTELLAR) registry” of STEMI patients aged 20 years or older treated by primary PCI. The INTERSTELLAR registry is a retrospective, observational, four regional hospital based registry that reflects current management practices, risk factors, and clinical outcomes in patients with STEMI undergoing primary PCI in the cities of Incheon and Bucheon located in the mid-western part of the Korean peninsula between 2007 and 2014 (clinicaltrials.gov identifier NCT02804958) [11, 12]. A well-trained study coordinator collected data using a standard protocol. The Institutional Review Boards of the four participating hospitals approved the study protocol, which also complied with the Declaration of Helsinki (6th revision) and written consent was obtained from each patient. Patients previously diagnosed with coronary artery disease, cardiomyopathy, valvulopathy (≥moderate), pericardial disease, or congenital heart disease were excluded (n = 71), as were patients with established liver disease, bone disease, or chronic kidney disease capable of affecting ALP levels or clinical outcomes (n = 275), and patients that lacked an initial serum ALP level test result (n = 13). Chronic kidney disease was defined as an estimated glomerular filtration rate of < 60 mL/min/1.73 m^2^, as determined using the 4-variable Modification of Diet in Renal Disease formula. After applying the above-mentioned inclusion and exclusion criteria, 1178 (82.7% male, mean age 58.2±12.3 years) of the originally considered 1537 patients were included in the present study.

### Demographic and laboratory data

Demographic data, cardiovascular risk factors, and laboratory data were available. Baseline blood testing was performed and recorded as is routine for patients that visited the emergency rooms with a complaint of chest pain and in whom acute coronary syndrome was suspected.

### Primary PCI

All procedures were performed according to current standard guidelines. Before PCI, patients were pre-medicated with aspirin (at least 100 mg), and a loading dose of P2Y12 receptor antagonist was administered. Heparin was administered throughout the procedure in order to maintain an activated clotting time of 250 seconds or longer. A glycoprotein IIb/IIIa receptor blocker was administered at the discretion of the operator. Coronary angiography was performed using standard techniques. Decisions to use thrombectomy devices, intravascular ultrasound, an intra-aortic balloon pump, and percutaneous cardiopulmonary support were made by the operator. Procedural success was defined as no in-hospital death, no emergency bypass surgery, and the achievement of Thrombolysis in Myocardial Infarction (TIMI) flow grade 3 in the distal portion of the infarct-related artery and the presence of <30% residual stenosis.

### Follow-up

After primary PCI, all patients were monitored in a coronary care unit for at least 24 hours. Two-dimensional transthoracic echocardiography was performed within 12 hour of the index procedure. Standard medical management was provided by responsible physicians. The primary study outcome was a major adverse cardiac or cerebrovascular event (MACCE), defined as the composite of all-cause death, non-fatal myocardial infarction, non-fatal stroke, and ischemia-driven revascularization. Patient follow-up data were collected using review of all medical records and/or standardized telephone interviews.

### Statistical analysis

Continuous variables are presented as means ± standard deviations for normally distributed data or as medians (interquartile ranges) for skewed data. Categorical variables are described using absolute and relative (percentage) frequencies. Patients were divided into tertiles by initial serum ALP level (<64, 65–82, and >83 IU/L), and these three groups were compared with respect to baseline characteristics and adverse clinical outcomes using one-way analysis of variance for continuous variables or using Pearson’s χ^2^ test for categorical variables. Survival rates were estimated using the Kaplan-Meier product-limit estimation method with the log-rank test. Multivariate Cox regression analysis was performed to quantify relationships between covariates previously reported to be related with adverse outcomes or ALP levels and time to event and tertiles of ALP level. Restricted cubic spline regression, adjusted for the same covariates as in the fully adjusted Cox model, was used to explore associations between ALP levels and clinical outcomes. P values of less than 0.05 were considered statistically significant, and the analysis was performed using SPSS version 20 (SPSS, Chicago, IL, USA) and SAS version 9.4 (SAS Institute, Cary, NC, USA).

## Results

### Baseline characteristics

The demographic and laboratory data measured at time of presentation are shown in Table 1 by ALP level tertiles. ALP levels ranged from 14 to 192 IU/L (median 73 IU/L, interquartile range 61–79 IU/L). Most of the 1178 patients (n = 1120, 95.1%) had a serum ALP level below the upper limit of normal (reference value range, 35–123 IU/L). ALP values of the three tertiles were < 64 IU/L, 65 to 82 IU/L, and > 83 IU/L. The patients in the 65–82 and >83 IU/L tertiles tended to be older and female, and to have higher diastolic blood pressures and heart rates at time of presentation. However, neither risk factor profiles nor severities of MI, such as, Killip class, presence of anterior MI, or peak levels of creatine kinase-myocardial band isoenzyme, were significantly different among the tertiles. In addition, estimated glomerular filtration rate, serum phosphate, and serum hepatic enzymes were no different in the tertile groups.

**Table 1:** Demographic and laboratory data.

	ALP Tertile 1 < 64 IU/L (n = 391)	ALP Tertile 2 65–82 IU/L (n = 392)	ALP Tertile 3 > 83 IU/L (n = 395)	p value
***Demographic data***				
Age, years	57.3±12.6	58.1±12.5	59.3±12.0	0.066
Men, n (%)	342 (87.5)	330 (84.2)	302 (76.5)	<0.001
Body mass index, kg/m^2^	24.3±2.9	24.4±3.3	23.8±3.4	0.021
Diabetes mellitus, n (%)	82 (21.0)	88 (22.4)	95 (24.1)	0.586
Hypertension, n (%)	158 (40.4)	174 (44.4)	176 (44.6)	0.415
Systolic blood pressure, mm Hg	124.3±26.4	127.7±27.6	128.2±28.1	0.093
Diastolic blood pressure, mm Hg	75.8±17.1	78.1±17.1	79.4±18.0	0.014
Heart rate, per minute	75.1±19.2	77.6±18.3	78.5±19.2	0.034
Killip class >II, n (%)	31 (7.9)	35 (8.9)	40 (10.2)	0.544
Anterior wall MI, n (%)	203 (52.2)	197 (50.8)	216 (55.2)	0.442
***Laboratory data***				
Albumin, d/dL	4.17±0.37	4.24±0.38	4.26±0.42	0.003
Glucose, mg/dL	163.6±69.5	171.3±79.3	175.2±77.1	0.090
Total bilirubin, mg/dL	0.66±0.29	0.66±0.31	0.61±0.28	0.008
AST, IU/L	30 (22–57)	30 (22–57)	34 (24–58)	0.120
ALT, IU/L	27 (18–40)	26 (19–40)	27 (19–43)	0.653
Hypoxic liver injury^†^, n (%)	74 (18.9)	75 (19.2)	80 (20.3)	0.874
ALP, IU/L	56 (50–61)	73 (69–77)	97 (89–109)	<0.001
Creatinine, mg/dL	0.96±0.17	0.95±0.19	0.92±0.18	0.003
Estimated GFR, mL/min/1.73 m^2^	86.5±19.0	88.6±19.6	88.6±25.1	0.586
Calcium, mg/dL	8.84±0.52	8.95±0.54	8.99±0.55	<0.001
Phosphate, mg/dL	3.15±0.93	3.10±0.85	3.25±2.26	0.398
Initial CK-MB, ng/mL	4.5 (2.1–25.6)	5.0 (2.1–32.9)	5.9 (2.2–27.2)	0.383
Peak CK-MB, ng/mL	161.4 (60.8–283.2)	176.4 (75.5–300,0)	165.1 (69.7–300.0)	0.235

AST, aspartate aminotransferase; ALT, alanine aminotransferase; ALP, alkaline phosphatase; GFR, glomerular filtration rate; CK-MB, creatine kinase-myocardial band isoenzyme; hs-CRP, high-sensitivity C-reactive protein

^†^Hypoxic liver injury was defined as an elevation of serum transaminase level more than twice the upper limit of normal [13].

A summary of angiographic, procedural, and echocardiographic data are provided in Table 2. Although door-to-balloon times were slightly different, infarct-related artery, extent of coronary artery disease, baseline and final TIMI flow, and rate of procedural success were similar for the groups. In addition, left ventricular ejection fractions were similar in the three groups.

**Table 2:** Angiographic, procedural and echocardiographic data.

	ALP Tertile 1 < 64 IU/L (n = 391)	ALP Tertile 2 65–82 IU/L (n = 392)	ALP Tertile 3 > 83 IU/L (n = 395)	p value
***Angiographic and procedural data***				
Infarct related artery, n (%)				
Left main	4 (1.0)	2 (0.5)	3 (0.8)	NA
Left anterior descending	199 (51.2)	195 (50.3)	213 (54.5)	
Left circumflex	45 (11.6)	52 (13.4)	34 (8.7)	
Right coronary	141 (36.2)	139 (35.8)	141 (36.1)	
Extent of coronary artery disease, n (%)				
1-vessel	173 (44.6)	168 (43.3)	156.(39.8)	0.119
2-vessel	124 (32.0)	139 (35.8)	124 (31.6)	
3-vessel	91 (23.5)	81 (20.9)	112 (28.6)	
Baseline TIMI flow grade, n (%)				
0–2	353 (90.5)	358 (92.0)	358 (91.1)	0.752
3	37 (9.5)	31 (8.0)	35 (8.9)	
Final TIMI flow grade, n (%)				
0–2	35 (9.0)	46 (11.8)	41 (10.4)	0.435
3	354 (91.0)	343 (88.2)	352 (89.6)	
Door-to-balloon time, min	71 (57–88)	74 (58–88)	68 (58–85)	0.048
Symptom-to-balloon time, min	205 (133–397)	208 (126–380)	212 (135–404)	0.596
Procedural success, n (%)	354 (91.0)	343 (88.2)	352 (89.6)	0.435
***Echocardiographic data***				
LVEF, %	49.2±11.3	49.6±11.7	49.0±11.5	0.736
LVEDD, mm	51.5±5.4	54.4±5.9	50.2±4.6	0.044
LAVI, ml/m^2^	18.6±8.1	19.5±9.1	18.7±7.3	0.644
E/E’	11.2±4.4	11.6±5.3	11.9±4.9	0.466

### Association between the tertiles of ALP level and adverse clinical outcomes

**Fig 1:**
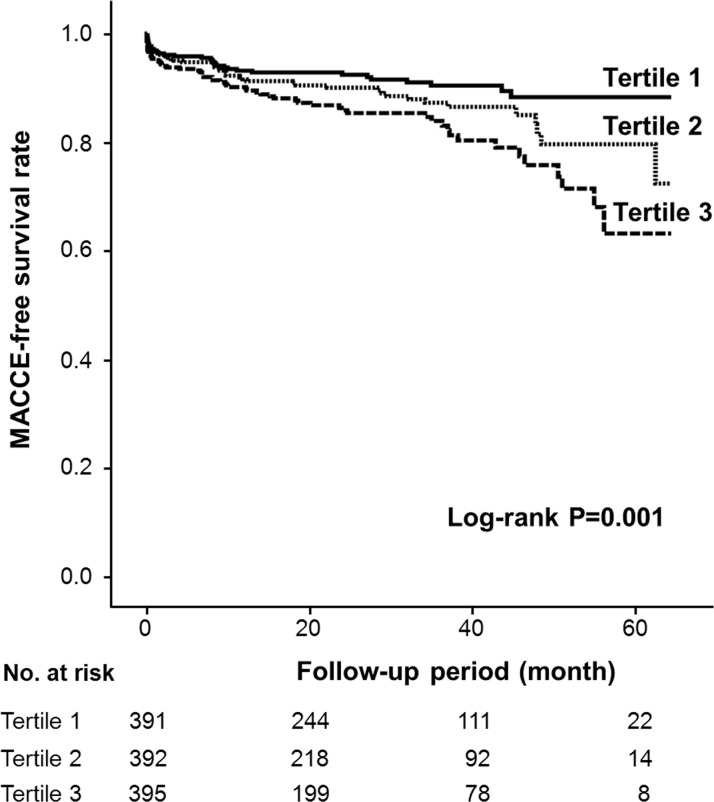
Major adverse cardiac or cerebrovascular event (MACCE)-free survival curve according to serum alkaline phosphatase level tertiles.

The incidences of adverse events are summarized in Table 3. Median duration of follow-up was 25 months (interquartile range, 10–39 months). Rate of MACCE significantly increased by tertiles. Of the composite of adverse events, non-fatal MI more frequently occurred in the higher than in the lower ALP tertile. Among 36 patients with non-fatal MI, 19 (52.8%) had non-ST-segment elevation MI, which more frequently occurred in the highest tertile than in the other tertiles (Tertile 1 vs. Tertile 2 vs. Tertile 3, 0.8% vs. 0.8% vs. 3.3%, respectively, p = 0.005). However, recurrence rate of STEMI was not significantly different between the 3 tertiles (0.8% vs. 1.8% vs. 1.8%, respectively, p = 0.391). Kaplan-Meier survival curves showed the higher ALP tertile experienced significantly more MACCE than the lower tertile as determined by the log-rank test (Fig 1).

**Table 3:** Incidence of adverse clinical outcomes according to the tertiles of ALP levels.

	ALP Tertile 1 < 64 IU/L (n = 391)	ALP Tertile 2 65–82 IU/L (n = 392)	ALP Tertile 3 > 83 IU/L (n = 395)	p for Trend
MACCE	34 (8.7)	46 (11.7)	62 (15.7)	0.003
All-cause death	18 (4.6)	20 (5.1)	26 (6.6)	0.221
Non-fatal myocardial infarction	6 (1.5)	10 (2.6)	20 (5.1)	0.004
Ischemia-driven revascularization	8 (2.0)	10 (2.6)	12 (3.0)	0.378
Non-fatal stroke	2 (0.5)	6 (1.5)	4 (1.0)	0.487

MACCE, major cardiac and cerebrovascular event

**Fig 2:**
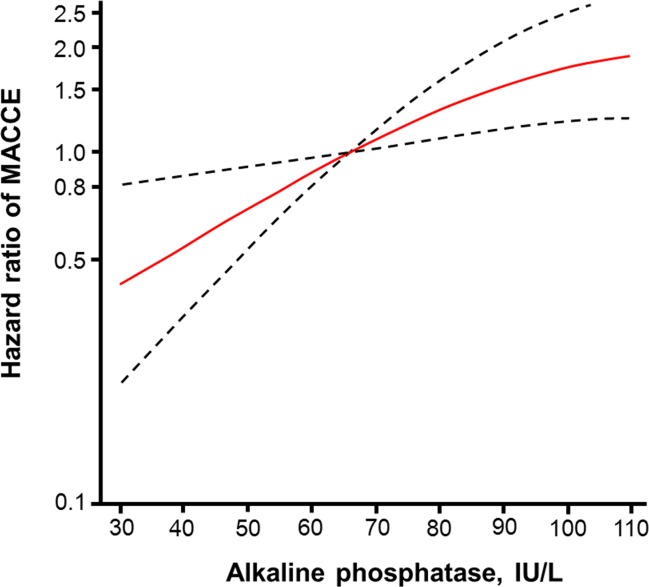
Restricted cubic spline regression model of serum alkaline phosphatase (ALP) levels for a major adverse cardiac or cerebrovascular event (MACCE). Hazard ratios (HRs) were adjusted for the variables using in Model 2 on Table 4. Knots were placed at the 10^th^, 50^th^, and 90^th^ percentiles of the ALP distribution (at 52 IU/L, 73 IU/L, and 103 IU/L, respectively). The solid line presents HR and dotted line 95% confidence interval of HR.

After adjusting for possible confounders, the adjusted hazard ratio (HR) of MACCE increased with tertile [adjusted HR (95% confidence interval), Tertile 1: reference, Tertile 2: 1.69 (1.01–2.81), Tertile 3: 2.46 (1.48–4.09), p for trend <0.001, Table 4]. Restricted cubic spline regression analysis suggested an approximately linear increase in the risk for MACCE as ALP level increased (Fig 2).

**Table 4:** Predictive value of alkaline phosphatase (ALP) for major adverse cardiac and cerebrovascular event (MACCE).

	Model 1^†^			Model 2^‡^		
	HR	95% CI	p value	HR	95% CI	p value
ALP						
Tertile 1	Reference			Reference		
Tertile 2	1.54	0.96–2.47	0.074	1.69	1.01–2.81	0.045
Tertile 3	2.15	1.36–3.39	0.001	2.46	1.48–4.09	<0.001

HR, hazard ratio; CI, confidence interval.

^†^Model 1: HRs have been adjusted for age, sex, diabetes mellitus, hypertension, ejection fraction and Killip class.

^‡^Model 2: HRs have been adjusted for Model 1 variables and additional covariates as follows: multi-vessel disease, anterior wall myocardial infarction, symptom to balloon time, estimated glomerular filtration rate, peak creatine kinase-myocardial band isoenzyme, serum aspartate aminotransferase, serum alanine aminotransferase, albumin, total bilirubin, glucose, calcium, and phosphate.

We also analyzed associations between ALP tertile groups and MACCE in different subgroups. No significant interaction was found between the risk of MACCE and a higher ALP tertile in the presence of; diabetes (p for interaction = 0.901), anterior wall MI (p = 0.917), multivessel disease (p = 0.574), left ventricular systolic dysfunction (ejection fraction < 40%, p = 0.155), or hypoxic liver injury (serum transaminase level > twice the upper limit of normal, p = 0.263).

## Discussion

In the present study, most patients (about 95%) with STEMI that underwent primary PCI had a serum ALP level below the upper limit of normal. Serum ALP levels, measured at time presentation, were found to be independently associated with the risk of MACCE after successful primary PCI in STEMI patients. To the best of our knowledge, this study is the first study to report an association between serum ALP at presentation and cardiovascular events in patients with STEMI undergoing primary PCI. Furthermore, it extends previous findings regarding acute MI requiring primary PCI. Based on these results, we suggest serum ALP level at presentation in the emergency room setting might serve as an early prognostic marker in patients with STEMI.

ALP is actually enzyme found primarily liver and bone, and to lesser extents in intestine, placenta, and kidneys. In clinical practice, serum ALP levels are usually used to diagnosis bone or liver diseases, such as, renal osteodystrophy or cholestasis. However, it has been suggested ALP plays a pivotal role in mineral metabolism and might be a biochemical marker of vascular calcification [7, 8, 14]. Furthermore, recent publications have shown a significant association exists between elevated ALP levels and cardiovascular events and mortality in various populations, such as, hemodialysis patients, survivors of stroke or MI, and the elderly [7–10]. Park et al. demonstrated that an elevated ALP level predicted mortality, MI, and stent thrombosis after PCI in 1,636 coronary artery disease patients, and suggested this was mediated by its promotion of coronary calcification [8]. However, the patients recruited in this previous study ranged from stable angina to STEMI patients, and acute MI patients constituted only ~20% of the cohort. It is also known that coronary calcification is less frequently observed in STEMI patients than in those with other coronary artery diseases [15]. In the present study, we sought to evaluate the association between serum ALP levels and adverse clinical outcomes in STEMI patients with a relatively low calcification burden. Our findings suggest that serum ALP levels measured in an emergency room may be useful for predicting adverse events, especially non-fatal re-infarction in STEMI patients, although notably, most of these patients (about 95%) had ALP levels below the upper limit of normal.

The underlying mechanisms responsible for this association between elevated ALP levels and the increased risk of adverse events in patients with acute MI are unclear. The first possibility concerns a putative link between ALP and vascular calcification. ALP inactivates inorganic pyrophosphate, which is a potent inhibitor of hydroxyapatite crystal growth and a potential local and circulating inhibitor of vascular calcification [5, 14]. Indeed, in one study ALP up-regulation was observed in vessels with medial calcification [16], and in another, an independent association was found between ALP and coronary calcification in hemodialysis patients [17]. In turn, vascular calcification could be associated with poor clinical outcomes mediated by its deleterious effects on plaque stability, vascular stiffness, valvular heart disease, and calciphylaxis [14]. The extent of coronary artery calcification correlated with plaque burden [18], and could predict future cardiovascular events including mortality or MI [19]. In this study, non-fatal MI was more frequently occurred in the higher tertile than in the lower tertile, which suggested that higher ALP level might be related with higher magnitude of plaque burden or coronary artery calcification. However, because data on the severity of vascular calcification or bone-specific ALP were not available in this study, this mechanism remains speculative.

The second possibility is that the association between ALP and adverse events could be affected by another confounder, such as, liver disease or inflammation. Liver enzyme abnormalities are commonly observed in acute MI, possibly due to hypoxic liver damage [20], and it recently was suggested that hypoxic liver injury (defined as serum transaminase level more than twice the upper limit of normal) is closely associated with post-PCI left ventricular dysfunction and mortality in STEMI [13]. ALP levels are also slightly elevated in the presence of hypoxic liver injury, which can lead to confounding of the prognostic effect of ALP. However, in this study, ALP levels were not significantly different according to the Killip class or the presence of hypoxic liver injury, although hypoxic liver injury was more frequently observed in the high Killip class (III-IV) than in the low class (I-II) (29.2% vs. 18.4%, respectively, p = 0.007). In hypoxic liver injury by shock, serum transaminase was more sensitive than biliary enzymes, such as bilirubin or ALP. The elevation of ALP level was more prominent in congestive hepatopathy with elevated filling pressure than in ischemic hepatopathy with low cardiac output [21]. Thus, this finding suggests that hypoxic livery injury is caused by acute left-heart failure, resulting in acute elevation of serum transaminase levels with no significant difference of ALP level according to hemodynamic status at time of the presentation in STEMI patients. In addition, in this study, preexisting liver disease was excluded and other liver enzymes were adjusted for during the analysis. Thus, we believe significant confounding by hypoxic liver injury was unlikely.

Inflammatory status might be another confounder, as ALP may be associated with markers of inflammation, which would be expected to adversely influence outcomes [22, 23]. C-reactive protein levels within 6 hours after the onset of acute MI were found to be associated with adverse coronary events [24]. However, in coronary artery disease the correlation between ALP and C-reactive protein level was minimal [8]. Furthermore, previous studies conducted in several populations have shown that serum ALP independently predicts outcome (after adjusting for C-reactive protein) [8–10]. In the present study, no information was available on inflammatory status, and thus, this hypothesis requires further study.

Some caveats of the present study should be considered. First, we cannot preclude the possibility of residual confounding. To overcome this limitation, patients with chronic renal failure, and therefore, abnormal bone metabolism, were excluded and multivariate analysis was adjusted for multiple potential confounders known to be related with ALP levels. Second, since serum ALP levels were examined only during the acute period of MI, MI severity might have influenced ALP levels. For this reason, we adjusted the analysis for confounders, such as, Killip class, anterior wall MI, and left ventricular dysfunction, known to influence short-term complications in the setting of acute MI. In our opinion, MI severity would not have influenced our findings considerably because the exclusion of in-hospital mortality did not change the association between ALP and adverse events after MI. However, residual confounding could be yet another issue. Third, serum ALP levels were not measured using a central laboratory. Fourth, the mechanism underlying the association between ALP levels and adverse events remains to be elucidated. Finally, the observation nature of this study precludes conclusions regarding the causality of the association between ALP and adverse outcomes.

## Conclusion

Our results demonstrate that increased serum ALP at time of presentation, though below the upper limit of normal, were independently associated with a higher risk of adverse cardiac or cerebrovascular events after acute MI requiring primary PCI. This result suggests serum ALP be viewed as a potential marker of the burden of vascular disease. Further studies are required to clarify the possibility of a causal link between high ALP, vascular calcification and adverse vascular events.
